# Probiotics‐derived postbiotics improve host metabolic syndrome by activating the adipocyte thermogenic signaling pathway in high‐fat diet‐fed mice

**DOI:** 10.1002/imo2.70089

**Published:** 2026-03-05

**Authors:** Ye Tian, Liujing Huang, Yifan Liu, Xudong Mai, Yanbo Cheng, Bingdong Liu, Yixin Chen, Liwei Xie

**Affiliations:** ^1^ Faculty of Synthetic Biology Shenzhen University of Advanced Technology Shenzhen China; ^2^ State Key Laboratory of Applied Microbiology, Southern China, Guangdong Provincial Key Laboratory of Microbial Culture Collection and Application, Guangdong Open Laboratory of Applied Microbiology, Institute of Microbiology Guangdong Academy of Sciences Guangzhou China; ^3^ Department of Endocrinology and Metabolism, Zhujiang Hospital Southern Medical University Guangzhou China; ^4^ Department of Rehabilitation Shenzhen Children's Hospital Shenzhen China; ^5^ Fuyang Institute of Zhejiang Chinese Medical University Hangzhou China

**Keywords:** *Bifidobacterium*, Indole‐3‐acetic acid (IAA), *Lactobacillus*, metabolic syndromes, postbiotics, synthetic biology

## Abstract

Metabolic syndrome, including type 2 diabetes mellitus (T2DM) and obesity, represents a global health challenge. While probiotics can modulate host metabolism, postbiotics (inanimate microbial preparations) have emerged as potentially safer and more stable therapeutic alternatives. The current study investigates the effects of postbiotics derived from five human‐origin bacterial strains in mice model with high‐fat diet‐induced metabolic syndrome. Using multi‐omics profiling, we demonstrate that supplementation of postbiotics significantly ameliorates weight gain, improves glucose tolerance and insulin sensitivity, and alleviates hepatic steatosis. Transcriptomic analysis indicates that the treatment upregulated adipocyte genes related to thermogenesis and fatty acid oxidation while downregulating hepatic lipid synthesis. Most importantly, the intervention reshapes the gut microbiota structure. Target metabolomics reveals that this microbial remodeling is accompanied by a significant elevation of indole‐3‐acetic acid (IAA), a tryptophan‐derived microbial metabolite, in the host serum. Functional studies in primary adipocytes confirm that IAA is sufficient to trigger thermogenic gene expression and activate AMP‐activated protein kinase (AMPK) signaling. These findings suggest a mechanism where postbiotics improve metabolic health by modulating the gut ecosystem to enrich beneficial metabolites like IAA, which in turn ameliorates the metabolic syndrome and promotes adipocyte thermogenesis and lipolysis. The present research supports postbiotics as a promising therapeutic strategy for metabolic syndrome and highlights the importance of the gut microbiota‐metabolite axis.

## Highlights

- Postbiotics derived from novel *Lactobacillus* and *Bifidobacterium* strains ameliorate metabolic syndrome and induce adipocyte thermogenesis in high‐fat diet‐fed mice.
- Postbiotics intervention reshapes the gut microbiota structure and modulates the host‐microbe metabolic interaction landscape to reverse diet‐induced dysbiosis.
- The microbiota‐associated metabolite indole‐3‐acetic acid (IAA) is enriched by postbiotics treatment and is sufficient to trigger thermogenic signaling in primary adipocytes.

## Graphical abstract

This schematic illustrates how probiotics‐derived postbiotics (PLBK®1–5) combat high‐fat diet‐induced obesity. Postbiotics remodel gut microbiota, boosting beneficial metabolites. These metabolites activate adipocyte thermogenesis (Ucp1/AMPK pathway), resulting in reduced body weight, improved insulin sensitivity, and alleviated hepatic steatosis in the treated mouse model.

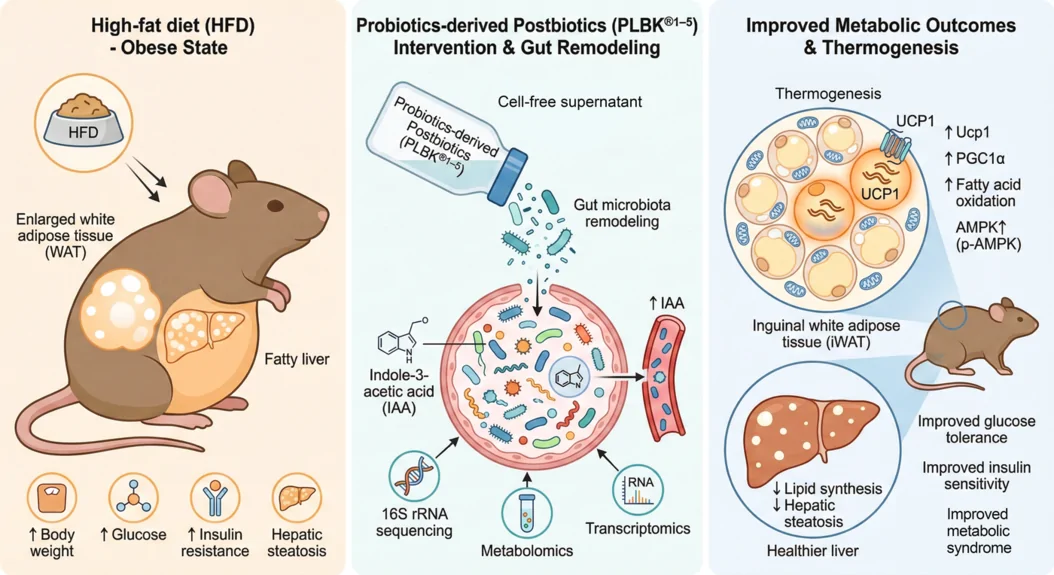

## 1 INTRODUCTION

Metabolic syndrome includes a cluster of interconnected physiological, biochemical, and metabolic factors that directly increase the risk of cardiovascular disease and type 2 diabetes mellitus (T2DM) [1, 2, 3]. It is clinically characterized by the presence of at least three of the following conditions: abdominal obesity, elevated blood pressure, hyperglycemia, hypertriglyceridemia, and reduced high‐density lipoprotein cholesterol [1, 2, 3]. The global prevalence of metabolic syndrome has reached epidemic proportions, affecting 20%–25% of the adult population worldwide, with significant variations across different ethnicities, age groups, and geographical regions.

Probiotics, defined as live microorganisms that confer health benefits when administered in adequate amounts, have emerged as potential therapeutic agents for metabolic syndromes. Clinical studies have demonstrated that specific strains of *Lactobacillus* and *Bifidobacterium* can modulate glucose homeostasis, lipid metabolism, and inflammatory pathways [4, 5, 6]. For instance, administration of *Lactobacillus reuteri* has been shown to improve insulin sensitivity and reduce systemic inflammation. Similarly, *Lactobacillus gasseri* supplementation has demonstrated efficacy in reducing visceral adiposity and improving lipid profiles through modulation of bile acid metabolism and adipokine production. Meta‐analyses of randomized controlled trial (RCT) have confirmed that probiotic interventions significantly reduce body mass index, waist circumference, fasting plasma glucose, and glycated hemoglobin levels in patients with metabolic syndrome [7, 8, 9, 10].

However, the clinical application of live probiotics faces significant challenges, including the risk of systemic infections in immunocompromised individuals, the potential for acquiring antibiotic resistance genes, and difficulties in maintaining viability during manufacturing, storage, gastrointestinal transit, and potential acholic and drug administration [11]. Recent advances in microbiome research have shifted attention toward postbiotics‐defined as preparations of inanimate microorganisms and/or their components that confer a health benefit on the host [12]. Postbiotics offer several advantages over live probiotics, including enhanced stability, safety, and standardization of bioactive compounds [13]. These attributes make postbiotics an attractive stand‐alone therapeutic strategy for metabolic disorders, potentially decoupling the beneficial effects from the risks associated with live bacterial administration.

The metabolic benefit of postbiotics is mediated through multiple mechanisms, including restoration of intestinal barrier function, modulation of gut microbial composition, and the secretion of bioactive metabolites that regulate host metabolic pathways [13]. Short‐chain fatty acids (SCFAs), particularly acetate, propionate, and butyrate, represent the most extensively studied postbiotics metabolites with established roles in glucose and lipid metabolism [14, 15]. SCFAs enhance insulin sensitivity through activation of G‐protein coupled receptors (GPR41, GPR43) and stimulation of glucagon‐like peptide‐1 secretion, while simultaneously promoting fatty acid oxidation and inhibiting lipogenesis in the liver [16, 17]. Emerging evidence suggests that other microbial metabolites, particularly tryptophan derivatives, play critical roles in host–microbe communication and energy homeostasis. Specifically, indole‐3‐acetic acid (IAA), a tryptophan metabolite produced by gut bacteria, has been recently linked to improved insulin sensitivity and reduced hepatic steatosis in metabolic disease models [18, 19].

The present study was designed to investigate the therapeutic potential of probiotics‐derived postbiotics in improving systemic glucose, lipid, and energy metabolism in an experimental model of metabolic syndrome. Specifically, we utilized postbiotics derived from five novel strains (*Lactobacillus reuteri* PLBK®1 and PLBK®2, *Lactobacillus gasseri* PLBK®3, *Lactobacillus acidophilus* PLBK®4, and *Bifidobacterium lactis* PLBK®5) that were originally isolated from the feces of healthy human donors and selected for their probiotic potential. Rather than comparing these fractions to live bacteria, the present study aims to evaluate the postbiotics preparation as a defined intervention to determine if cell‐free components alone are sufficient to drive metabolic improvements. Using a comprehensive multi‐omics approach, we identified that postbiotics intervention reshapes the gut microbiota and enriches specific metabolites, such as IAA. We further provide evidence that this alteration is associated with activated thermogenesis in adipocytes and enhanced lipolysis in hepatocytes. These findings provide mechanistic insights into the metabolic benefits of postbiotics and suggest a novel therapeutic strategy for the management of metabolic syndrome.

## 2 RESULTS

### 2.1 Integrated genomic, metabolomic, and functional profiling of novel probiotic strains

In the present investigation, we aimed to comprehensively characterized postbiotics prepared by utilizing the five probiotic strains, *Lactobacillus reuteri* PLBK^®️^1 and PLBK^®️^2, *Lactobacillus gasseri* PLBK^®️^3, *Lactobacillus acidophilus* PLBK^®️^4, and *Bifidobacterium lactis* PLBK^®️^5 through multiple biological approaches, including genomics, metabolomics, transcriptomics, and in vivo functional profiling, as outlined in the experimental workflow (Figure 1A). This included not only single‐strain cultures and characterization but also investigations into multi‐strain interactions and subsequent in vivo evaluation using an animal model to assess the functional effects of the probiotics‐derived postbiotics (Figure 1A). Genomic sequencing and annotation revealed a core set of genes across all strains related to metabolism and cellular processes (Figure 1B). Importantly, phylogenetic analysis demonstrated that these represented five novel strains, exhibiting distinct genetic distances from previously characterized strains within their respective species, suggesting unique genomic characteristics and potentially novel functional properties (Figure S1). This genomic foundation was further expanded by targeted metabolomics of culture supernatants from the single‐strain cultures (Figure 1A), which identified distinct metabolite profiles for each strain (Figure 1C, full metabolite profiles provided in online Supplementary Data). The major classes of metabolites were further categorized (Figure 1D), suggesting functional differences in their metabolic outputs and potential host‐interaction mechanisms. Notably, while diverse organic acids and amino acids were abundant, IAA was not detected at detectable levels in the cell‐free fermentation supernatants of the individual PLBK® strains under the in vitro culture conditions. These in vitro findings provide a basis for the subsequent in vivo experiments, linking the novel metabolomic characteristics and combinations to observed functional effects.

**Figure 1:**
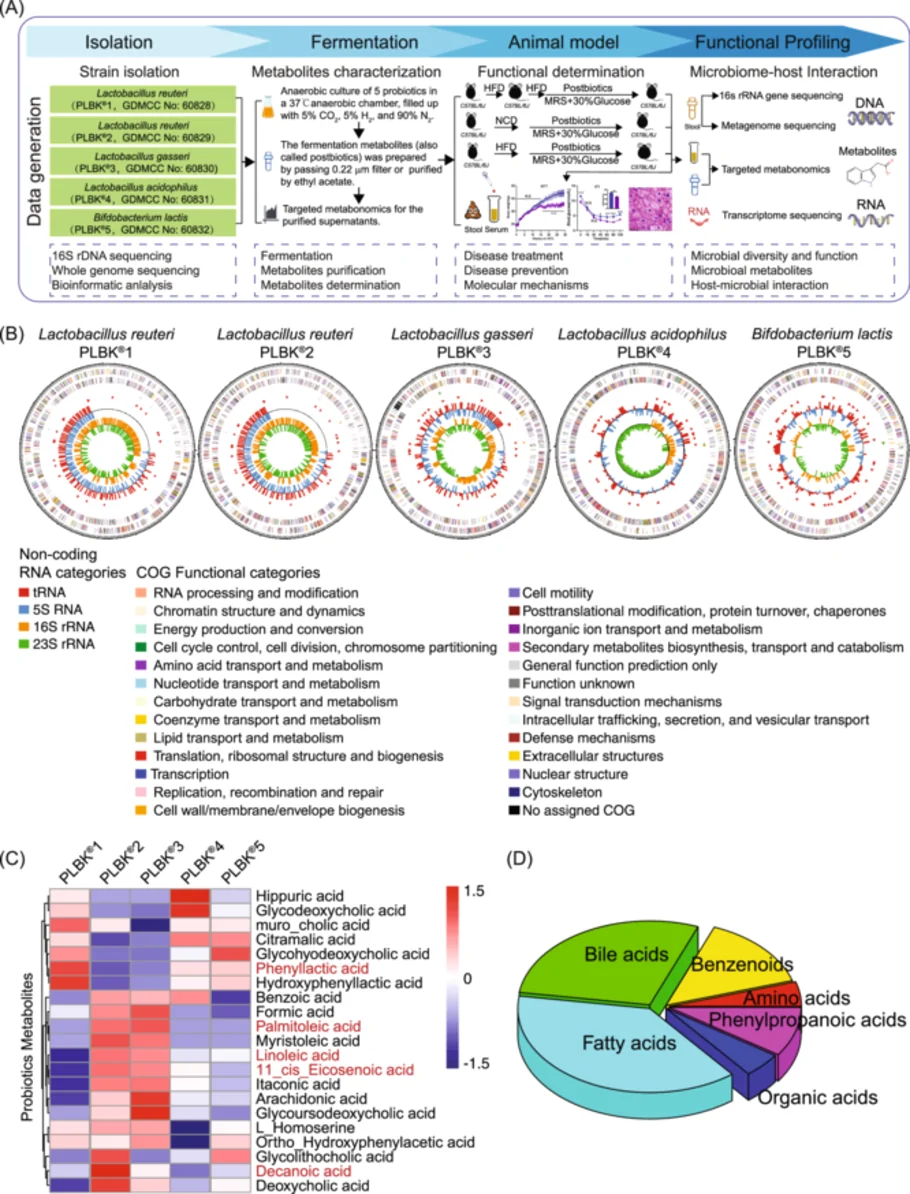
Isolation, fermentation, and functional characterization of probiotic strains and their metabolites. (A) Schematic workflow of the research pipeline comprising four sequential phases: isolation, fermentation, animal model testing, and functional profiling. From left to right: isolation phase involved five probiotic strains (PLBK®1‐5); fermentation phase included metabolite characterization, postbiotic preparation, and targeted metabolomics; animal model phase evaluated functional effects under normal chow diet (NCD) and high‐fat diet (HFD) conditions; functional profiling phase incorporated microbiome–host interaction analyses. Bottom panel displays methodologies employed at each phase, including 16S rDNA sequencing, whole genome sequencing, bioinformatics analysis, metabolite purification and identification, transcriptomics, and targeted metabolomics. (B) Comparative genomic circular maps of five probiotic strains: *Lactobacillus reuteri* (PLBK®1 and PLBK®2), *Lactobacillus gasseri* (PLBK®3), *Lactobacillus acidophilus* (PLBK®4), and *Bifidobacterium lactis* (PLBK®5). The concentric tracks, from the outermost to the innermost, display: (i) Coding sequences (CDS) on the forward and reverse strands, color‐coded according to COG functional classifications (e.g., cell motility, energy production, amino acid transport, and carbohydrate metabolism; see bottom legend for full color key); (ii) Non‐coding RNA genes, depicted as triangular arrowheads indicating transcriptional direction and color‐coded by category: tRNA (red), 5S RNA (blue), 16S rRNA (orange), and 23S rRNA (green); and (iii) The central histograms representing GC content and GC skew, indicating variations in nucleotide composition across the genome. (C) Heatmap depicting metabolite profiles produced by the five probiotic strains. Vertical axis represents probiotic strains, horizontal axis displays detected metabolites, including hippuric acid, glycodeoxycholic acid, phenyllactic acid, benzoic acid, and palmitoleic acid. (D) Pie chart illustrating the distribution of major metabolite categories produced by the probiotic strains. Metabolites are classified into six principal groups: bile acids, benzenoids, amino acids, phenylpropanoic acids, organic acids, and fatty acids.

### 2.2 Postbiotics improve metabolic dysfunction in the diet‐induced obese mice model

The metabolic benefit was further demonstrated in mice model with supplementation of postbiotics in drinking water on a normal chow diet (NCD) or high‐fat diet (HFD) feeding induced an obese phenotype for 12 weeks (Figure 2A). Compared to the H_2_O group, postbiotics supplementation in the following 12 weeks significantly ameliorated the body and tissue weight gain, improved glucose tolerance, insulin sensitivity on both dietary feeding (Figures 2B–D, S2A–C), and serum lipid profiles with lower TCHO, TG, LDL‐c, AST, and ALT levels (Figure 2E). Phenotypical images and histological examinations revealed the decreased size of adipose tissues and liver, with a marked reduction in hepatic lipid droplet accumulation (Figure 2F,G), correlated with significantly lower liver TG level (Figure S2D). Hepatic gene expression profiling via RNAseq (Figure 2H) revealed that postbiotics downregulated a cluster of genes (Cluster I) enriched for steroid biosynthesis, lipid biosynthesis regulation, cholesterol efflux, and adipose tissue development (Figures 2I, S2E,F), including the TGFβ and PPAR signaling pathways.

**Figure 2:**
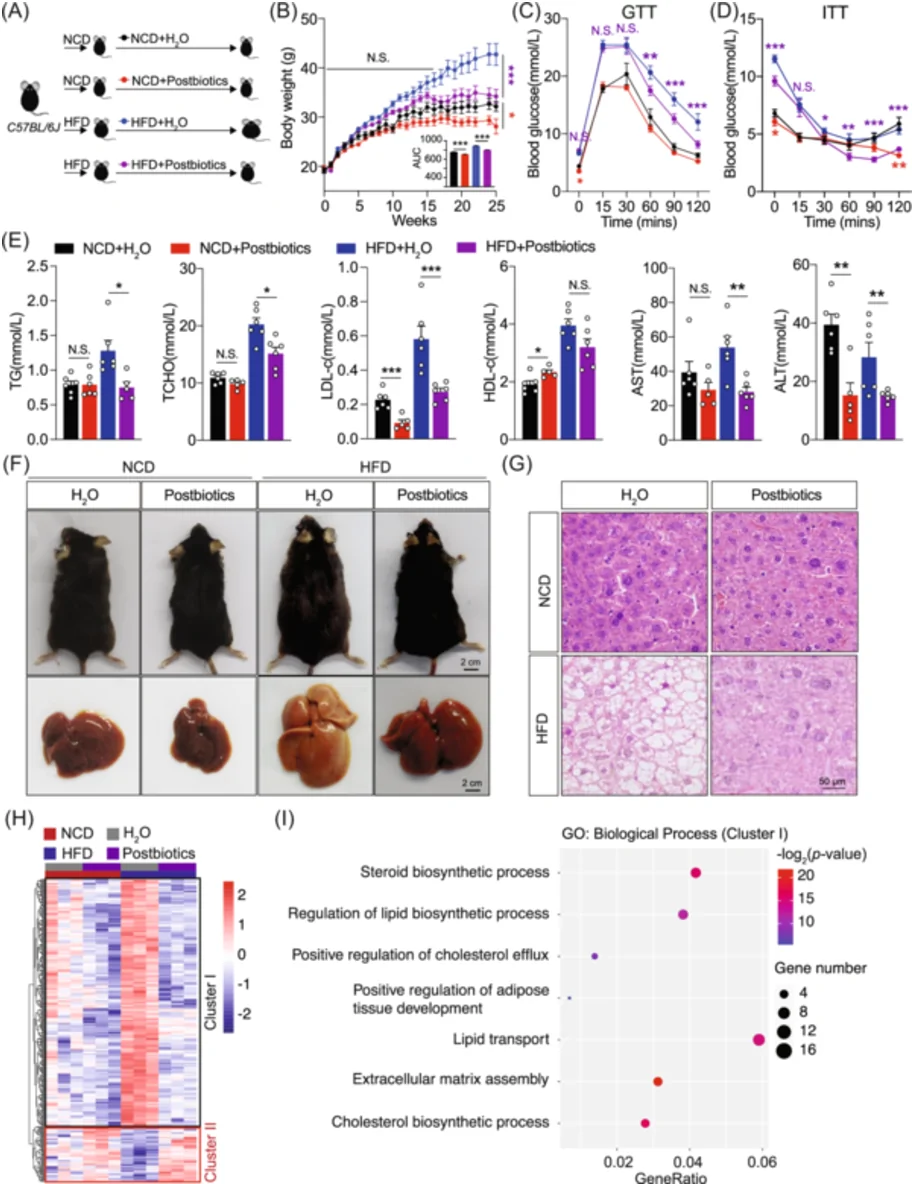
Effects of probiotic‐derived postbiotics on high‐fat diet‐induced obesity and metabolic dysfunction. (A) Experimental design schematic illustrating four treatment groups: chow diet with water (NCD + H_2_O), chow diet with postbiotics (NCD + Postbiotics), high‐fat diet with water (HFD + H_2_O), and high‐fat diet with postbiotics (HFD + Postbiotics). *C57BL/6J* mice were used as the experimental model (*n* = 8–10 per group). (B) Body weight gain over 25 weeks across all experimental groups. Data are presented as mean ± SEM, and comparison was made between H_2_O and Postbiotics weekly for each time‐point for NCD and HFD, respectively, via the multiple unpaired *t*‐test, followed by multiple comparisons using Holm–Šídák's. Area under the curve (AUC) analysis of changes in body weight over 25 weeks. Purple (HFD) and red (NCD) asterisks denote statistical comparison between H₂O and Postbiotics groups, respectively, for body weight at Week 25. (C) Glucose tolerance test (GTT) results showing blood glucose levels (mmol/L) at 0, 15‐, 30‐, 60‐, 90‐, and 120‐ min post‐glucose administration. Purple (HFD) and red (NCD) asterisks denote statistical comparison between H₂O and Postbiotics groups via the multiple unpaired *t*‐test, followed by multiple comparisons using Holm–Šídák's. (D) Insulin tolerance test (ITT) results displaying blood glucose levels (mmol/L) at 0, 15‐, 30‐, 60‐, 90‐, and 120‐min following insulin injection. Purple (HFD) and red (NCD) asterisks denote statistical comparison between H₂O and Postbiotics groups via the multiple unpaired *t*‐test, followed by multiple comparisons using Holm–Šídák's. (E) Serum biochemical parameters analysis, including triglycerides (TG), total cholesterol (TCHO), low‐density lipoprotein cholesterol (LDL‐c), high‐density lipoprotein cholesterol (HDL‐c), aspartate aminotransferase (AST), and alanine aminotransferase (ALT) levels. (F) Representative images of morphology and liver appearance. The upper panel shows whole‐body photographs of mice from each treatment group; the lower panel displays corresponding liver photographs demonstrating macroscopic differences in hepatic tissue. (G) Liver histological analysis by H&E staining. Scale bar: 50 μm. Images reveal pronounced hepatic steatosis in the HFD + H_2_O group (lower left) that was attenuated by postbiotics treatment (lower right). (H) Heatmap of hepatic transcriptome analysis illustrating differential gene expression profiles across the four experimental groups. Color gradient from blue (downregulated) to red (upregulated) represents relative gene expression levels. Genes are clustered into two distinct groups (Cluster I and Cluster II). (I) Gene Ontology (GO) analysis of enriched biological processes in Cluster I, including steroid biosynthetic process, regulation of lipid biosynthetic process, positive regulation of cholesterol efflux, positive regulation of adipose tissue development, lipid transport, extracellular matrix assembly, and cholesterol biosynthetic process. Bubble size represents gene number, color intensity indicates −log_2_(*p*‐value), and the *x*‐axis shows gene ratio. Data were presented as mean ± SEM, with individual data points representing individual mice. *p < 0.05, **p < 0.01, ***p < 0.001, N.S. indicates no significant difference.

### 2.3 Postbiotics activate thermogenic pathways in adipose tissue against HFD‐induced metabolic dysfunction

Histological analysis of interscapular brown adipose tissue (iBAT) and inguinal white adipose tissue (iWAT) revealed that postbiotics treatment altered fat cell morphology in HFD‐fed mice (Figure 3A). Specifically, in iWAT, postbiotics significantly reduced the mean area of oil bubbles compared to the H_2_O group for both NCD and HFD feeding (Figure 3B). Immunohistochemical staining for Ucp1 and PGC1α in iWAT showed significantly higher levels of Ucp1 and PGC1α upon postbiotics treatment in the HFD group (Figure 3C). Principal coordinate analysis (PCoA) of gene expression profiles in iWAT demonstrated that the Postbiotics group clustered distinctly from the H_2_O group, and more closely with the NCD diet‐fed groups (Figure 3D). A heatmap of differentially expressed genes presented two major clusters (Figure 3E). Genes in Cluster I were upregulated by postbiotics and involved in cellular lipid catabolic processes, brown fat cell differentiation, fat cell differentiation, lipid oxidation, and fatty acid oxidation (Figure 3F). Those in Cluster II downregulated by postbiotics were enriched for genes related to the regulation of lipid biosynthetic processes, fatty acid biosynthetic processes, regulation of lipid storage, and lipid localization (Figure 3G). These findings indicate that postbiotics enhance thermogenic adipocyte differentiation and lipid oxidation, reduce lipid accumulation, and modulate the expression of genes involved in lipid metabolism.

**Figure 3:**
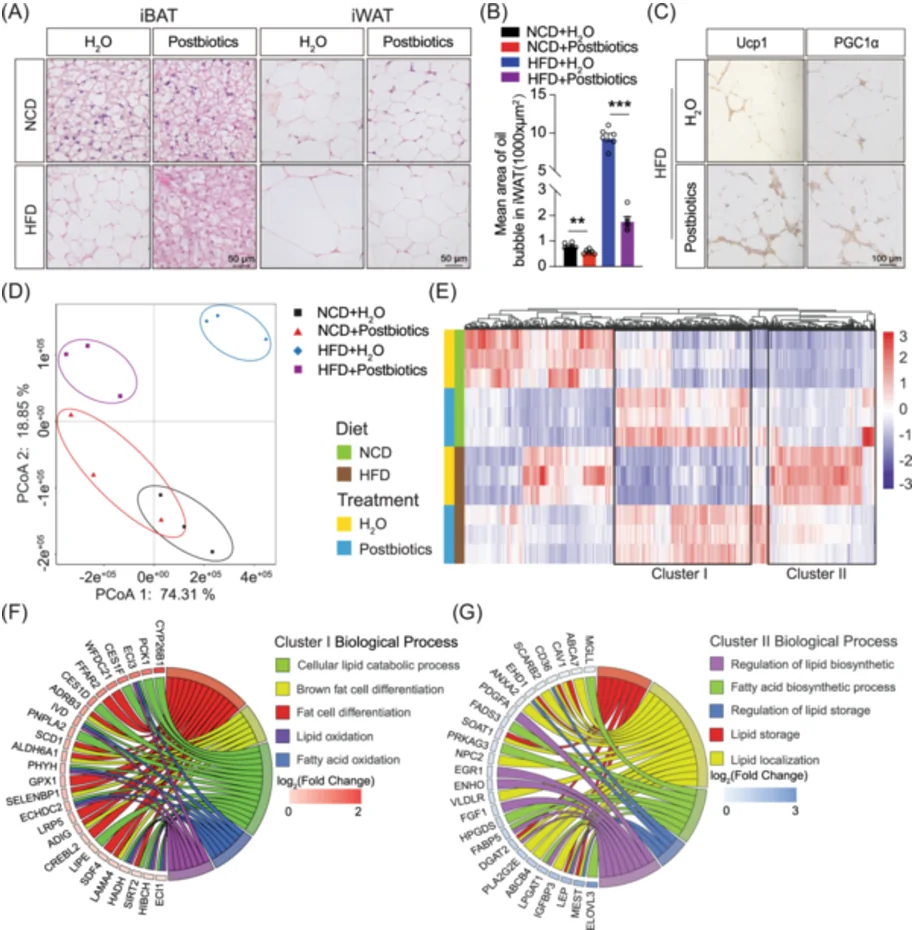
Effects of postbiotics supplementation on adipose tissue phenotype and transcriptomic signatures in diet‐induced obesity. (A) Representative histological sections of interscapular brown adipose tissue (iBAT) and inguinal white adipose tissue (iWAT) from mice fed chow (NCD) or high‐fat diet (HFD), with or without postbiotics supplementation. H&E staining reveals morphological differences across treatment groups. Scale bars: 50 μm. (B) Quantification of mean lipid droplet area (μm²) in iWAT across four experimental groups. (C) Immunohistochemical analysis of thermogenic markers Ucp1 and PGC1α expresssion in iWAT from HFD‐fed mice with or without postsbiotic treatment. Scale bar: 100 μm. (D) Principal Coordinate Analysis (PCoA) of iWAT transcriptome data showing distinct clustering of experimental groups. PCoA1 accounts for 74.31% of variation, while PCoA2 explains 18.85% of variation, demonstrating clear separation based on both diet type and postbiotics treatment. (E) Heatmap visualization of differentially expressed genes in iWAT across experimental groups. Color scale represents normalized expression values (red: upregulated; blue: downregulated). Hierarchical clustering identified two major gene clusters (Cluster I and Cluster II) with distinct expression patterns. (F) Chord diagram depicting enriched biological processes in Cluster I genes, including cellular lipid catabolic process, brown fat cell differentiation, fat cell differentiation, lipid oxidation, and fatty acid oxidation. Gene connections are shown between specific genes and their associated biological processes. Color intensity represents log_2_ (Fold Change). (G) Chord diagram illustrating enriched biological processes in Cluster II genes, including regulation of lipid biosynthetic process, fatty acid biosynthetic process, regulation of lipid storage and lipid localization. Gene connections are shown between specific genes and their associated biological processes. Color intensity represents log_2_ (Fold Change). Data presented as mean ± SEM. Statistical significance: **p < 0.01, ***p < 0.001 compared to respective controls.

### 2.4 Postbiotics improve glucose homeostasis, reduce hepatic steatosis, and prevents HFD‐induced metabolic disorders

*C57BL/6J* mice were fed with HFD concurrently with the treatment with either H_2_O or Postbiotics (Figure 4A). The postbiotics significantly ameliorated the body weight gain over the 30‐week period (*p* < 0.05) (Figure 4B). Representative images of the mice showed a visually apparent reduction in overall size in the Postbiotics group compared to the H_2_O group (Figure 4C). The ratio of tissue/body weight of iWAT and eWAT was significantly decreased, despite no change for iBAT and Liver (Figure S3A,B). Postbiotics treatment significantly improved glucose clearance (at 60, 90, and 120 min) and insulin sensitivity (Figures 4D,E, S3C,D). Liver was observed with a paler color in the H_2_O group, indicative of lipid accumulation, and this was less pronounced in the Postbiotics group (Figure 4F, left). Histological analysis of liver sections stained with H&E confirmed reduced lipid droplet number and size with postbiotics treatment (Figure 4F, middle). Oil‐Red O staining, which specifically stains neutral lipids, further demonstrated a significant reduction in hepatic triglyceride (TG) content in the Postbiotics group (Figure 4G, right). However, there were no significant changes in serum levels of TG, TCHO, HDL‐c, LDL‐c, NFEA, AST, and ALT in the Postbiotics group (Figure S3E–K). Histological analysis indicated that postbiotics activated thermogenesis in both iBAT and iWAT (Figure 4H,I). These results demonstrate that postbiotics improve glucose homeostasis and reduce hepatic steatosis in mice with diet‐induced obesity, associated with the activation of thermogenesis and improved systemic energy metabolism.

**Figure 4:**
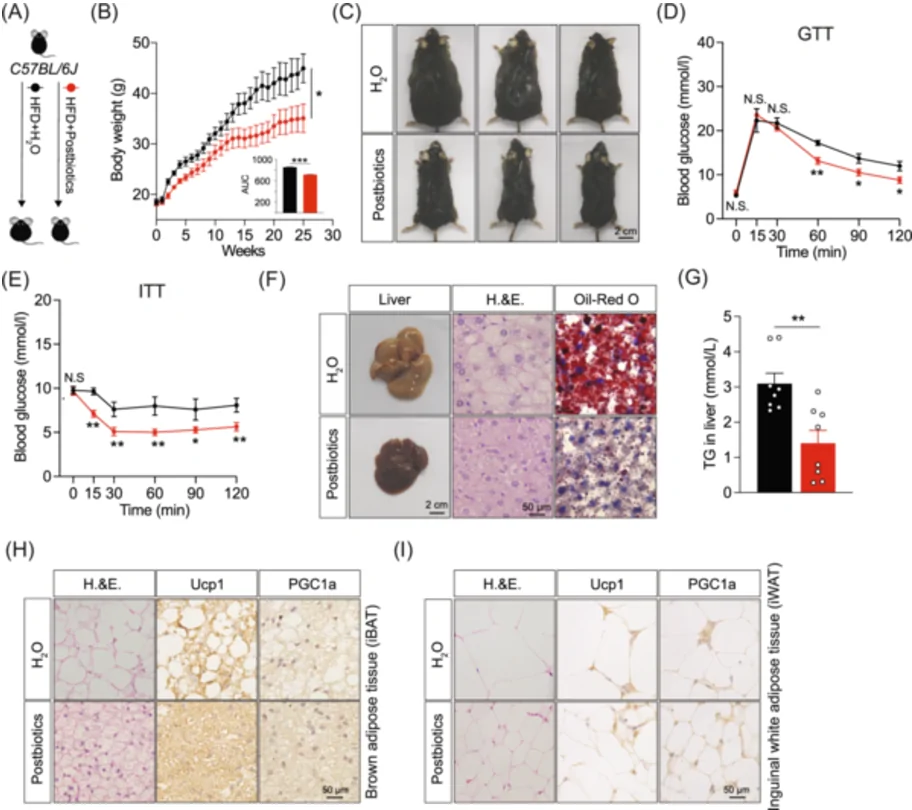
Postbiotics supplementation attenuates obesity and metabolic abnormalities in HFD‐fed mice. (A) Schematic representation of the experimental design using *C57BL/6J* mice fed a high‐fat diet (HFD) with either water (HFD + H_2_O, black) or postbiotics supplementation (HFD + Postbiotics, red). (B) Body weight was measured over 25 weeks of HFD intervention. Mice receiving postbiotics exhibited significantly reduced weight gain compared to the control group. The asterisks denote statistical comparison between the H₂O and Postbiotics groups, respectively, for body weight at Week 25. Significance was analyzed via the multiple unpaired *t*‐test, followed by multiple comparisons using Holm–Šídák's. Area under the curve (AUC) analysis of changes in body weight over 25 weeks. (C) Representative photographs of mice from both experimental groups (top: H₂O; bottom: Postbiotics) demonstrating visibly reduced adiposity in postbiotic‐treated animals. (D) Glucose tolerance test (GTT) results showing blood glucose concentrations (mmol/L) at baseline and following intraperitoneal glucose administration (0–120 min). Postbiotic‐treated mice displayed significantly improved glucose clearance at later time points. Asterisks denote statistical comparison between H₂O and Postbiotics groups via the multiple unpaired *t*‐test, followed with multiple comparisons using Holm–Šídák's (E) Insulin tolerance test (ITT) results showing blood glucose concentrations (mmol/L) following insulin challenge (0–120 min). Postbiotics supplementation significantly enhanced insulin sensitivity throughout the test period. Asterisks denote statistical comparison between H₂O and Postbiotics groups via the multiple unpaired *t*‐test, followed by multiple comparisons using Holm–Šídák's. (F) H&E‐stained liver sections (50 μm scale bar) demonstrating decreased lipid accumulation with postbiotics treatment; Oil Red O‐stained liver sections confirming reduced hepatic lipid content following postbiotics supplementation. (G) Quantification of hepatic triglyceride (TG) content (mmol/L) showing significantly lower levels in postbiotic‐treated mice. (H) Interscapular brown adipose tissue (iBAT) analysis with H&E staining and immunohistochemical detection of thermogenic markers Ucp1 and PGC1α. Postbiotics treatment increased multilocular adipocyte morphology and enhanced expression of thermogenic proteins. Scale bar: 50 μm. (I) Inguinal white adipose tissue (iWAT) analysis with H&E staining and immunohistochemical detection of thermogenic markers Ucp1 and PGC1α. Postbiotics supplementation promoted browning of white adipose tissue as evidenced by increased expression of thermogenic markers. Scale bar: 50 μm. Data presented as mean ± SEM. *p < 0.05, **p < 0.01, N.S. indicates no significant difference.

### 2.5 Postbiotics differentially shape gut microbiota composition and host metabolome

The influence of postbiotics on gut microbiota was assessed via the 16S rRNA sequencing and subsequent bioinformatic analysis. α‐diversity, measured by the Inverse Simpson index, showed no statistically significant differences between the Postbiotics and H_2_O groups, NCD and HFD feeding for 12 weeks on H_2_O followed by Postbiotics or H_2_O intervention for another 12 weeks (Figure 5A). However, β‐diversity analysis using principal component analysis (PCA) demonstrated clear separation for NCD but not for HFD groups based on gut microbiota composition (Figures S4A, 5B,C). Adonis analysis further confirmed that the clustering in the NCD group was statistically significant (*p* = 0.027), while that in the HFD group was not (*p* = 0.42). For mice models receiving postbiotics (P) or H_2_O (H) concurrently with the HFD, showed no significant difference on α‐diversity (Figure 5D), while PCA of the PHFD groups revealed distinct clustering, with significant differences along PC1 and PC2, suggesting that concurrent administration of postbiotics or H_2_O with the HFD also significantly altered microbial communities in both small intestine (HI) and feces (HF) (Figure S4B, Adonis *p *= 0.020 for Figure 5E, Adonis *p* = 0.014 for Figure 5F). Analysis of the relative abundance of bacterial phyla showed that while *Firmicutes* and *Bacteroidetes* were dominant phylum across all groups, subtle shifts in their relative abundances, along with changes in less abundant phylum like *Proteobacteria*, *Actinobacteria*, and others, likely contribute to the observed β‐diversity differences between the dietary groups and postbiotics treatments (Figure S4E and S4F). We utilized liquid chromatography‐mass spectrometry (LC‐MS) to probe the metabolites of fecal and serum samples of mice under NCD and HFD diets. Partial Least Squares Discriminant Analysis (PLS‐DA) of both fecal and serum metabolites further highlighted the impact of HFD and postbiotics intervention. PLS‐DA plots showed clear separation between the groups receiving H_2_O and postbiotics within both the NCD (Figure 5G,H) and HFD (Figure 5I,J) conditions, indicating distinct metabolic profiles both in feces and serum. The VIP scores from the PLS‐DA (Figure S4G and S4H) identified key metabolites driving these separations by volcano plots (Figure 5K,L). Further analysis using the dot plots (Figure 6A) and VIP score analysis (Figure 6B) highlighted key metabolites, such as indoleacetic acid and phenylpyruvic acid (increased with postbiotics) and acetylcarnitine and α‐linolenic acid (decreased with postbiotics), that consistently differentiate postbiotic‐treated groups from H_2_O, regardless of the type of diets. Other metabolites, like arachidonic acid, show diet‐dependent responses. Individual metabolite plots (Figure 6C,D) confirm the increase in IAA with postbiotics treatment. Volcano plots illustrated the differential abundance of specific metabolites in serum between the NCD groups receiving postbiotics (NCD_PS) or water (NCD_HS), and between the HFD groups receiving postbiotics (HFD_PS) or water (HFD_HS) (Figure 5K,L). Numerous metabolites, spanning various classes including amino acids, benzoic acids, bile acids, fatty acids, and others, were significantly altered. The changes in these metabolites' z‐scores (Figure 6E,F) between the indicated comparisons (e.g., NCD_PF vs. NCD_HF, representing fecal samples from the NCD groups receiving postbiotics vs. H_2_O, respectively, after the 12‐week dietary intervention) further quantified the metabolic shifts.

**Figure 5:**
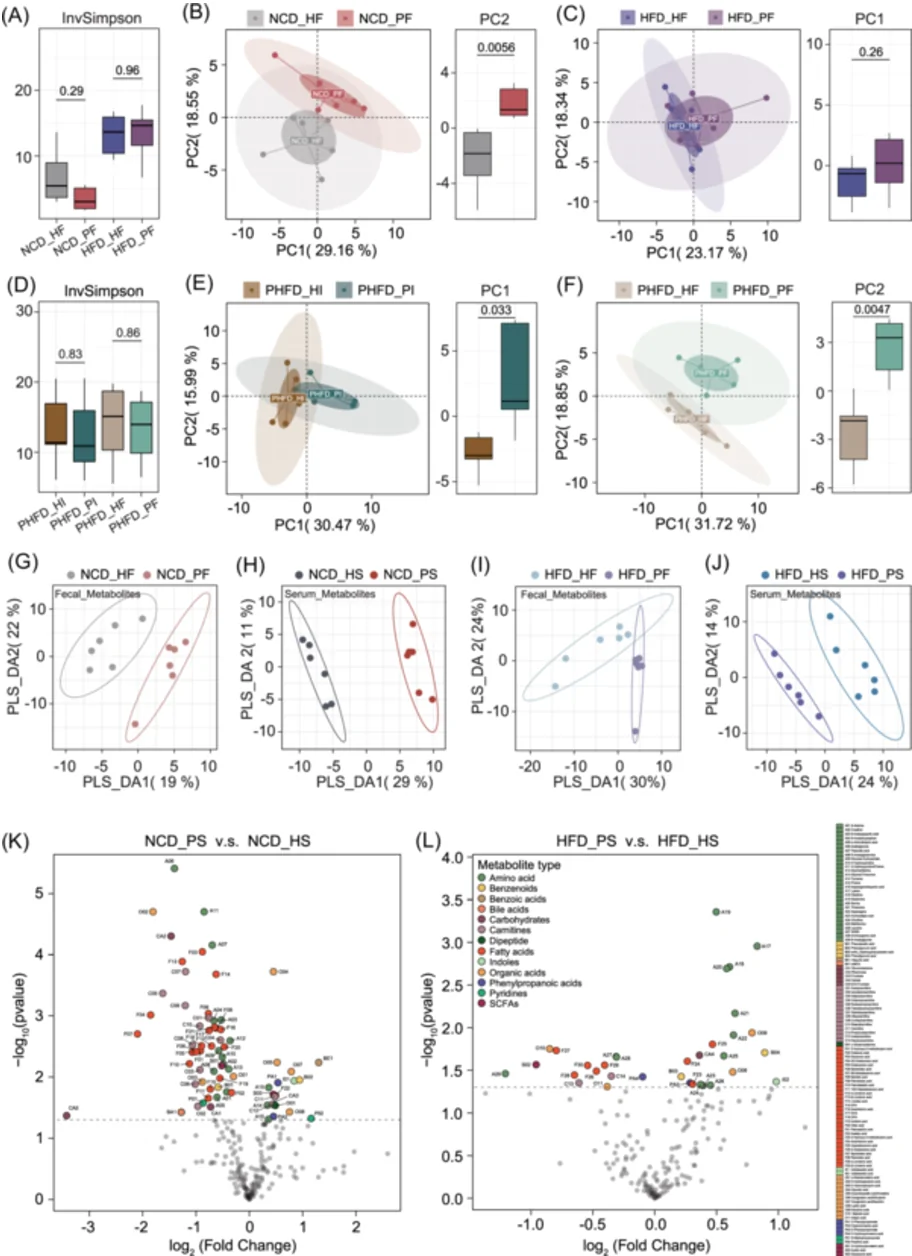
Postbiotics modify the structure of gastrointestinal flora and host metabolism under different dietary patterns. (A) Differences in alpha‐diversity between the postbiotics intervention group from control group of normal chow diet (NCD) and high‐fat diet‐induced obesity (HFD) models. The *y*‐axis refers to the inverse Simpson index. (B, C) PCA of NCD groups (B) and HFD groups (C) on the first two principal components was performed based on fecal microbiota at the species level. Ellipses represent 50% and 95% CI. (D) Differences in alpha‐diversity between the postbiotics early intervention group (PHFD_H) from control group (PHFD_P) under a high‐fat diet pattern. The *y*‐axis refers to the inverse Simpson index. PCA of PHFD groups on the first two principal components was performed based on the small intestinal microbiota (E) and fecal microbiota (F). Ellipses represent 50% and 95% CI. Partial least squares discriminant analysis (PLS‐DA) of fecal metabolites (G) and serum metabolites (H) in the NCD groups, and fecal metabolites (I) and serum metabolites (J) in the HFD groups. Ellipses in PLS‐DA represent a 95% CI. Statistical analysis was performed using Welch's *t*‐test (*n* = 6 per group). (K, L) Volcano plots depicting the effect of postbiotics in the serum metabolome by comparing the NCD_HS group versus the NCD_PS group (K), and the HFD_PS group versus the HFD_HS group (L). Differential metabolites are labeled. The extent of the log_2_‐transformed fold change value represents the log_2_‐ratio of metabolite content between groups.

**Figure 6:**
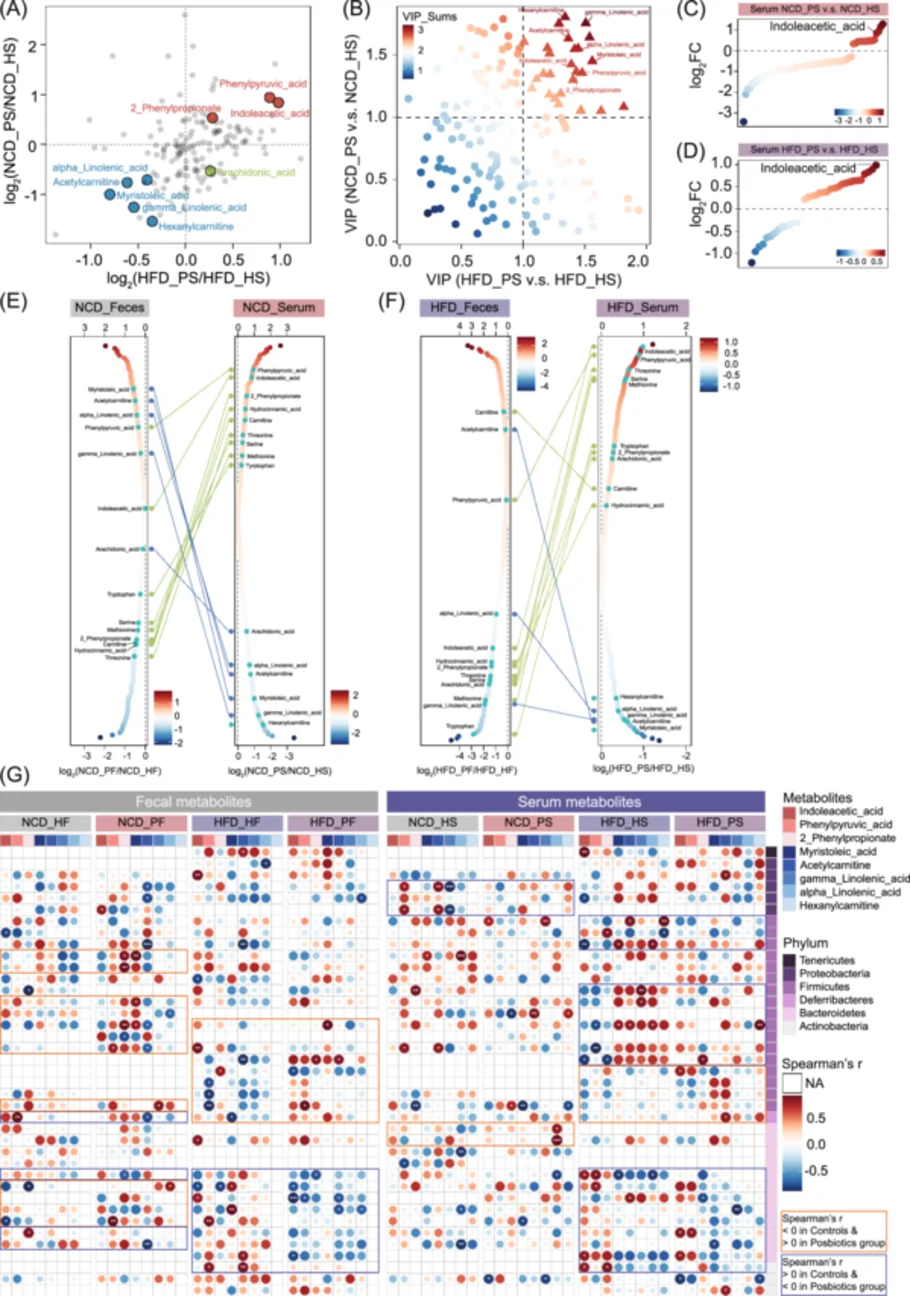
Integrated analysis of postbiotics‐related serum metabolites under different dietary patterns. (A) Upregulated (red) and downregulated (blue) metabolites in the serum of postbiotics intervention mice. The dot plot shows the metabolites in log_2_‐transformed fold change by comparing NCD_HS group versus NCD_PS group and HFD_HS group versus HFD_PS group. Metabolites with significant changes in both diet intervention models were colored by red, blue, and green (*p*‐value < 0.05). (B) Variable of importance in projection (VIP) scores of each metabolite used in the partial least squares discriminant analysis (PLS‐DA) models show constant significance of the postbiotics‐related serum metabolites. (C, D) The discriminatory metabolites were ranked according to the extent of the log_2_‐transformed fold change value by comparing NCD_PS group versus NCD_HS group (C), and HFD_PS group versus HFD_HS group (D). Indoleacetic acid significantly enriched in the serum under postbiotics intervention. (E, F) All metabolites were ranked according to the extent of the log_2_‐transformed fold change value by comparing NCD_PF group versus NCD_HF group and NCD_PS group versus NCD_HS group (E), and HFD_PF group versus HFD_HF group and HFD_PS group versus HFD_HS group (FK). The postbiotics‐related metabolites identified in PLS‐DA and discriminatory analysis were labeled with lake blue. Upregulated (green) and Downregulated (blue) metabolites in the serum of postbiotics intervention mice were presented with a line graph. (G) Heatmap illustrating Spearman's rank correlation coefficients between the relative abundance of species and discriminatory metabolites (*p < 0.05, **p < 0.01, ***p < 0.001). Only those bacterial species with annotation at genus level were included in the Spearman correlation analysis and shown in matched phylum level for each genus.

To investigate the interplay between gut bacteria and host metabolism under postbiotics intervention, we performed Spearman correlation analysis between the relative abundance of bacterial genera (annotated at the genus level with matched phylum) and the levels of key metabolites identified as significantly altered by postbiotics treatment (Figures 5K,L, and 6G). In the HFD_HF (H_2_O) group, genera under *Bacteroidetes* showed predominantly negative correlations with the key metabolites. Conversely, in both postbiotic‐treated groups (NCD and HFD), those genus under *Firmicutes* exhibited predominantly positive correlations with these metabolites, in both fecal and serum. Furthermore, outlined regions within the heatmap highlight specific, localized changes in gut microbiota–metabolite correlations induced by postbiotics, indicating a targeted rather than global effect. These findings demonstrate that postbiotics treatment significantly alters the relationships between specific bacterial taxa and key host metabolites, shifting the overall interaction landscape (Figure 6G). This effect is consistent across both different dietary groups, although the specific correlation patterns differ.

### 2.6 IAA is enriched in postbiotic‐treated mice and stimulates thermogenic gene expression in white adipocytes

Targeted metabolomic analysis of serum from mice treated with postbiotics revealed distinct metabolic profiles compared to the H_2_O group (Figure 7A). A Venn Diagram showed the number of metabolites significantly altered in the serum of NCD and HFD‐fed mice (Figure 7B). Among the metabolites significantly increased by postbiotics, IAA was the top candidate induced in serum of mice on both NCD and HFD (Figures 6E,F, and 7B). IAA was selected for further mechanistic exploration based on three criteria: (i) robust enrichment in the postbiotics intervention groups compared with controls, (ii) concordant elevation in both intestinal (feces) and circulating compartments (serum) (Figure 6E), and (iii) strong positive associations with systemic metabolic improvements. Quantification of serum IAA levels confirmed a significant elevation in both NCD and HFD‐fed mice treated with postbiotics (Figure 7C). To investigate the direct effects of IAA on adipocytes, primary white adipocytess were treated with increasing concentrations of IAA (0, 10, 15, and 25 µM). IAA treatment significantly increased the mRNA expression of *Ucp1*, *Cidea*, *Cox7a1*, and *Cox8b* in a dose‐dependent manner, while *Ppargc1α*, *Dio2*, and *Prdm16* were not significantly affected (Figure 7D). Western blot analysis of primary white adipocytes treated with IAA (0, 10, 15, and 25 µM) showed an increased protein level of Ucp1, PGC1α, and increased phosphorylation of AMPK (Figure 7E). These findings indicate that IAA, a metabolite enriched in the postbiotics treatment group, is sufficient to trigger a thermogenic program in white adipocytes that is associated with the activation of AMPK signaling.

**Figure 7:**
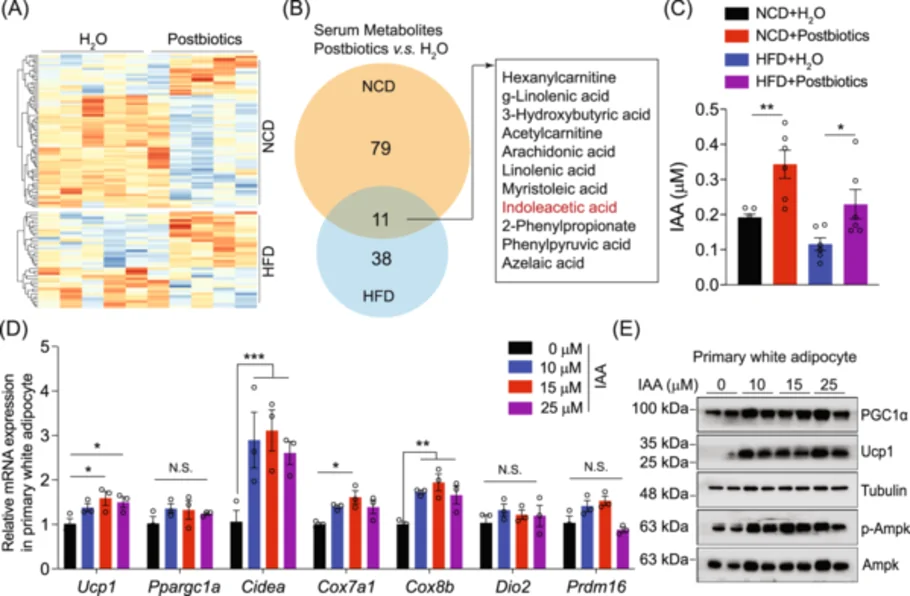
Indoleacetic acid (IAA) activates adipocyte beigeing and thermogenesis. (A) Heatmap of the differential metabolites level in serum of mice on rodent chaw and HFD between H_2_O and postbiotics group. (B) Overlapped serum metabolites between rodent chaw and HFD. (C) Serum IAA level of mice on rodent chaw and HFD between the H_2_O and postbiotics group. (D) qPCR analysis of gene expression (*Ucp1*, *Ppargc1α*, *Cidea*, *Cox7a*, *Cox8b*, *Dio2*, and *Prdm16*) in primary white adipocytes with treatment of IAA at different concentrations (0, 10, 15, 25 μm). (E) Western blot analysis of protein levels (PGC1α, Ucp1, Tubulin, p‐Ampk, and t‐Ampk) in primary white adipocytes with treatment of IAA at different concentrations (0, 10, 15, 25 μm). Data were presented as mean ± SEM. *p < 0.05, **p < 0.01, ***p < 0.001, N.S. indicates no significant difference.

## 3 DISCUSSION

Metabolic syndrome, characterized by obesity, T2DM, and cardiovascular risks, poses a significant global health challenge, affecting 20%–25% of children and adults worldwide [20, 21]. Microbial interventions, including probiotics and fecal microbiota transplantation (FMT), have shown promise in modulating host metabolism, yet their limitations in stability and safety have raised a strong interest in postbiotics, including non‐viable microbial cells, components, and metabolites with enhanced standardization and therapeutic potential [12, 22]. Here, we demonstrate that postbiotics derived from five novel strains of *Lactobacillus* and *Bifidobacterium* ameliorate HFD‐induced metabolic dysfunction in mice by enhancing adipocyte thermogenic signaling, reshaping gut microbiota, and enriching the beneficial microbial metabolite, for example, IAA. These findings reveal a novel mechanism underlying postbiotics benefits and demonstrate postbiotics as an alternative for alleviating metabolic disorders.

Our study shows that postbiotics supplementation significantly reduces HFD‐induced weight gain, enhances glucose tolerance and insulin sensitivity, and attenuates hepatic steatosis in mice. These metabolic improvements align with prior reports on probiotics, which reduce body mass index and fasting glucose in metabolic syndrome patients [23]. However, postbiotics offer distinct advantages, including reduced infection risk and improved shelf‐life stability [24]. Transcriptomic profiling revealed that upregulation of thermogenic and lipid oxidation genes (*Ucp1*, *Ppargc1α*) in adipose tissue, alongside downregulation of lipid biosynthesis and storage‐related genes in liver and adipose tissue. Histological and physiological data further confirmed a browning‐like phenotype in iWAT and increased systemic energy expenditure, indicating that postbiotics enhance thermogenesis, a critical process for combating obesity [25].

While microbial metabolites like SCFAs are known to induce thermogenic programs via G‐protein‐coupled receptors [26], our work identifies a distinct pathway involving IAA, expanding the understanding of microbial mediators of host energy homeostasis. IAA, a tryptophan‐derived bacterial metabolite, is the central finding from our metabolomic profiling and is significantly enriched in the serum of postbiotic‐treated mice. Functional studies demonstrated that IAA at micromolar level directly stimulates thermogenic gene expression (*Ucp1*, *Cidea*, *Cox7a1*, *Cox8b*) and is activated with increased phosphorylation of AMPK signaling in primary white adipocytes in a dose‐dependent manner. This level is comparable but 100 times lower than the reported level in several studies of fatty liver diseases and atherosclerosis [18, 19]. AMPK, a master regulator of energy homeostasis, promotes fatty acid oxidation and inhibits lipogenesis [27], and its activation in the presence of IAA likely underpins the observed improvements in systemic metabolism.

Regarding the origin of IAA, although specific probiotic strains can produce indole derivatives, our in vitro data indicated that the postbiotics strains used here do not produce a detectable level of IAA in cell‐free supernatant. Instead, 16S rRNA sequencing suggests that postbiotic supplementation remodels the gut ecosystem, increasing the abundance of indigenous bacteria capable of tryptophan metabolism. Canonical microbial pathways for IAA production involve the deamination or decarboxylation of tryptophan via enzymes such as tryptophanase and aminotransferases [28], which are widely distributed among commensal *Firmicutes* and *Bacteroidetes* [29, 30]. We hypothesize that postbiotic‐induced changes in the gut environment favor the expansion of these IAA‐producing taxa (e.g., specific *Clostridium* or *Bacteroides* species), leading to the elevated systemic IAA levels [17]. This “cross‐feeding” effect highlights postbiotics' ability to restore functional metabolic output in a dysbiotic gut microbiota without relying on the strain's direct metabolite production.

Beyond these direct effects, the broader context of tryptophan metabolism and its derivatives, including IAA, offers critical insights into their therapeutic potential for metabolic syndrome components such as obesity, T2D, and fatty liver diseases. Tryptophan, an essential amino acid, is metabolized through several pathways in the host and gut microbiota, including the kynurenine, serotonin, and indole pathways, each with distinct implications for metabolic health [31, 32, 33, 34]. Recent studies have increasingly highlighted the protective role of indole‐3‐acetic acid (IAA) in metabolic diseases. For instance, IAA has been shown to alleviate non‐alcoholic fatty liver disease (NAFLD) by modulating macrophage polarization and reducing hepatic inflammation [18, 19]. Similarly, microbiota‐derived IAA acts as a ligand for the aryl hydrocarbon receptor (AhR), promoting intestinal barrier integrity and reducing metabolic endotoxemia—a hallmark of T2D linked to gut dysbiosis [35, 36]. Our observation of ameliorated glucose tolerance and enhanced insulin sensitivity in postbiotic‐treated mice, coupled with elevated IAA levels, supports the hypothesis that IAA contributes to these benefits, potentially through AMPK signaling and crosstalk with AhR‐dependent pathways.

The indole pathway, predominantly driven by gut bacteria such as *Clostridium*, *Bacteroides*, and certain *Lactobacillus* and *Bifidobacterium* species, produces metabolites like IAA, indole‐3‐propionic acid (IPA), and IAA derivatives, which have been increasingly linked to host physiology and metabolism. IAA, in particular, is synthesized via the decarboxylation of tryptophan by bacterial tryptophanases or through oxidative deamination pathways, and its systemic circulation allows it to exert effects beyond the gut [28]. Our findings build on emerging evidence that tryptophan‐derived microbial metabolites play a protective role in metabolic syndrome. For instance, studies have shown that IAA and related indoles exhibit anti‐inflammatory and antioxidant properties, reducing gut inflammation—a key driver of obesity‐associated insulin resistance and NAFLD [19, 37]. In the context of obesity, IAA's stimulation of thermogenic genes in adipocytes, as observed in our study, suggests a direct mechanism for increasing energy expenditure, counteracting excessive lipid accumulation in WAT. This aligns with reports that indole derivatives can modulate lipid metabolism by activating pathways like peroxisome proliferator‐activated receptor gamma (PPARγ), which regulates adipocyte differentiation and energy balance [38].

In T2D, tryptophan metabolites, including IAA, have been associated with improved insulin sensitivity and glucose homeostasis. IAA may influence glucose metabolism by reducing systemic inflammation and oxidative stress to improve insulin resistance [35]. Additionally, the interaction between IAA and the aryl hydrocarbon receptor (AhR), a ligand‐activated transcription factor, may mediate anti‐diabetic effects by modulating immune responses and gut barrier integrity, thereby reducing metabolic endotoxemia—a hallmark of T2D linked to gut dysbiosis [39]. Our observation of ameliorated glucose tolerance and enhanced insulin sensitivity in postbiotic‐treated mice, coupled with elevated IAA levels, supports the hypothesis that IAA contributes to these benefits, potentially through AMPK activation and AhR‐dependent pathways.

Regarding fatty liver diseases, particularly NAFLD alongside obesity, tryptophan metabolism and IAA hold significant therapeutic promise. NAFLD, characterized by hepatic steatosis and often progressing to non‐alcoholic steatohepatitis (NASH), is closely associated with metabolic syndromes and gut microbiota dysbiosis [40]. Our data show that postbiotics intervention reduces hepatic lipid accumulation, which may be partially mediated by IAA. Consistent with our findings, recent work has demonstrated that IAA administration mimics the effects of intermittent fasting in reducing hepatic steatosis by influencing mitochondrial function [19]. Recent studies suggest that indole derivatives, including IAA, protect against hepatic steatosis by inhibiting lipogenesis and promoting fatty acid oxidation in hepatocytes, potentially through AMPK and AhR signaling [40]. Moreover, IAA's anti‐inflammatory effects could mitigate hepatic inflammation, a critical factor in NASH progression, by suppressing pro‐inflammatory cytokines like TNF‐α and IL‐6, as demonstrated in preclinical models [41]. The interplay between gut‐derived IAA and liver metabolism also points to the gut‐liver axis as a key target for postbiotics therapies, where IAA may strengthen gut barrier function, reducing the translocation of lipopolysaccharides that drive hepatic inflammation [42]. While our study did not directly assess IAA's effects on hepatocytes, the transcriptomic downregulation of lipid synthesis genes in the liver of postbiotic‐treated mice suggests an indirect or systemic role for IAA, warranting further necessary investigation into its hepatoprotective mechanisms.

The therapeutic potential of IAA in metabolic syndrome is further underscored by its ability to modulate multiple tissues and pathways. Beyond thermogenesis in adipocytes, IAA's systemic effects likely involve crosstalk between adipose tissue and liver, key players in energy homeostasis. However, challenges remain in understanding the precise microbial sources of IAA production in our postbiotics strains and the host receptors or transporters mediating its effects. The variability in tryptophan metabolism across individuals, influenced by diet, gut microbiota composition, and genetic factors, also suggests that personalized approaches may be necessary to harness IAA's benefits [43]. Nonetheless, our identification of IAA as a mediator of postbiotics effects opens new avenues for targeting tryptophan metabolism in metabolic syndrome, potentially through dietary interventions (e.g., tryptophan‐rich diets) or engineered postbiotics enriched for IAA production.

Postbiotics also reshape gut microbiota composition, with β‐diversity analysis revealing distinct microbial clustering in HFD‐fed mice treated with postbiotics, despite unchanged α‐diversity. Shifts in *Firmicutes* and *Bacteroidetes* abundance, alongside changes in *Proteobacteria* and *Actinobacteria*, suggest targeted microbial modulation, consistent with studies linking specific taxa to metabolic outcomes in obesity [44]. Spearman correlation analysis further showed altered host–microbe metabolite interactions, with Firmicutes genera positively correlating with key metabolites like IAA in postbiotic‐treated groups. This targeted effect on microbial ecology likely underpins the production of beneficial metabolites, supporting the role of gut microbiota as a determinant of host metabolic health [45]. Additionally, metabolomic profiling identified differential abundance of other metabolites (e.g., carnitine, α‐linolenic acid), which may synergize with IAA to enhance lipid metabolism [46]. These findings highlight postbiotics as mediators of gut‐liver‐adipose tissue crosstalk, a critical axis in metabolic syndrome.

Translational feasibility is a critical consideration for these findings. The serum IAA levels observed in mice with supplementation of postbiotics (approximately 0.3–0.4 µM) fall within the physiological ranges reported in healthy human populations, which typically range from 0.1 to 5 µM depending on diet and microbiome composition [28]. This suggests that the therapeutic concentrations are biologically achievable. Furthermore, postbiotics present fewer regulatory and safety hurdles than live biotherapeutics, as they eliminate the risks of bacterial translocation and infection. The stability of postbiotics formulations also allows for more precise dosing, making them a promising candidate for standardized clinical interventions [47].

## 4 CONCLUSION

In summary, our study elucidates a novel mechanism by which postbiotics improve metabolic syndrome through microbiota‐associated IAA‐mediated activation of adipocyte thermogenic signaling. By integrating multi‐omics profiling with functional validation, we provide a comprehensive framework for understanding postbiotics effects on host metabolism and energy expenditure. These results position postbiotics as a promising alternative to live probiotics for obesity, T2D, and NAFLD management, with potential applications in precision medicine.

## 5 LIMITATIONS OF THE CURRENT STUDY

Despite these insights, several limitations must be addressed. First, while IAA emerged as a key metabolite, the contributions of other postbiotics components, such as cell wall fragments or peptides, remain unexplored. Second, while we observed a strong correlation between reshaped microbiota, elevated IAA, and metabolic improvement, we did not perform FMT or use gnotobiotic models to definitively prove that the altered microbiota alone is sufficient to transfer the phenotype. Third, regarding the mechanism, although we demonstrated that IAA treatment triggers a thermogenic program and is associated with AMPK phosphorylation, we did not perform receptor‐knockout experiments (e.g., AhR or GPRs) to fully map the upstream signaling cascade. Fourth, our study utilized a complex postbiotics mixture without an IAA‐depleted control; we cannot rule out the contribution of other bioactive components such as SCFs or microbial peptides. Future research should prioritize metagenomic analysis to identify the specific IAA‐producing taxa enriched by postbiotics and employ receptor‐specific antagonists to delineate the precise molecular pathways.

## 6 METHODS

### 6.1 Animals

All animal studies were approved by the Institute of Animal Care and Use Committee (IACUC) of the Institute of Microbiology, Guangdong Academy of Sciences (GDAS) (Permission #: GT‐IACUC201704071). *C57BL6/J* mice were housed under a 12‐h/12‐h light and dark cycle (7 a.m.–‐7 p.m.) at 23 ± 2°C. At the age of 6–7 weeks old, *C57BL6/J* mice were fed with either normal rodent chow (NCD) or 60% HFD. Body weight was monitored and recorded weekly. The blood glucose and serum biochemistry were measured to determine the glucose tolerance, insulin sensitivity, and lipid profiles.

### 6.2 Primary adipocyte isolation and culture

iWAT from 4‐week‐old *C57BL/6J* mice was dissected and digested for 30‐min at 37°C in collagenase buffer. The adipose tissue suspension was passed through a 40 µm cell strainer and centrifuged at 1000 × *g* for 10‐min at room temperature to pellet the stromal vascular fraction (SVF). The SVF pellet was resuspended in DMEM growth medium (DMEM supplemented with 10% fetal bovine serum and 1% penicillin–streptomycin) and seeded into 24‐well plates. Once SVF cells reached full confluence, the medium was switched to induction medium (growth medium containing IBMX, dexamethasone, indomethacin, and rosiglitazone) for 3 days, followed by differentiation medium (growth medium containing insulin and T3) for an additional 7 days. More detailed procedures were performed as described before in our recent works [48, 49].

### 6.3 RNAseq and bioinformatic analysis

Total RNA samples extracted from the liver or iWAT of experimental mice were sequenced using a BGI‐SEQ. 2500 platform (Beijing Genomics Institute). The RNAseq data were processed and analyzed by utilizing standard pipeline as described before [50, 51, 52, 53].

### 6.4 Protein isolation and western blot

Animal tissues or cells were homogenized in RIPA buffer. Protein concentration was measured by using BCA Protein Assays (Thermo Fisher Scientific), and 30 μg of total protein was resolved on a 10% SDS‐PAGE, followed by detection of target proteins with specific antibodies (Ucp1: ab10983, PGC1α: AB3242, AMPK: #5831, p‐AMPK: #2523 and Tubulin: T6199).

### 6.5 RNA isolation and quantitative real‐time PCR (qPCR)

Total RNA was isolated from tissues or cells by utilizing the TRIzol™ reagent (Thermo Fisher). 1 μg total RNA was reverse transcribed to cDNA, which was applied to qPCR as described before [50, 51]. The sequence of primers for qPCR is listed in Table S1.

### 6.6 16S rRNA gene amplicon sequencing and bioinformatics analysis

Fecal samples were collected, and bacterial DNA was extracted for 16S rRNA sequencing, followed by bioinformatic analysis as described before [52, 54, 55, 56, 57, 58]. Briefly, the E.Z.N.A.® Stool DNA Kit (D4015, Omega, Inc., USA) was used to extract DNA from fecal samples following the manufacturer's instructions, and the V3‐V4 region of the 16S rRNA gene was amplified with primers 341F (5'‐CCTACGGGNGGCWGCAG‐3') and 805R (5'‐GACTACHVGGGTATCTAATCC‐3'). The PCR products were purified using AMPure XT beads and quantified by Qubit. The amplicon libraries were assessed for size and quantity using Agilent 2100 Bioanalyzer and the Library Quantification Kit for Illumina, respectively, before sequencing on the NovaSeq PE250 platform by LC‐Bio Technology Co. Ltd. in Hangzhou, Zhejiang Province, China. Paired‐end reads were assigned to individual samples using unique barcodes and trimmed by removing the barcode and primer sequences. FLASH was used to merge paired‐end reads. To obtain high‐quality clean tags, raw reads underwent quality filtering using fqtrim (v0.94), and chimeric sequences were removed using Vsearch software (v2.3.4) [59]. Clean reads were imported to QIIME2 (version 2022.2) for further analysis. After dereplication using DADA2 (a plugin in QIIME2), a feature table and feature sequence were obtained. The alignment of species annotation was performed using the QIIME feature‐classifier function against the gg‐13‐8‐99‐515‐806‐nb‐classifier. R (version 4.2.1) was used for downstream analysis. Based on the R package EasyMicroPlot [52] and EasyMultiProfiler [53], the contaminants (relative abundance < 0.1% and prevalence < 50% mice within any subgroups) were removed. Alpha and beta diversity analyses were performed, and visualization results were generated. A permutational multivariate analysis of variance (PERMANOVA) was performed using the adonis function from the vegan package (v2.7‐2). The analysis was based on the Chao distance metric, with a total of 999 permutations performed to test the statistical significance of the postbiotics intervention effect.

### 6.7 Target metabolomics and bioinformatics analysis

Fecal and serum samples were collected and prepared for target metabolomics to quantify the bacteria‐derived metabolites as described before [55]. In short, the liquid Chromatograph Mass Spectrometer technique was employed to generate metabolomics data from fecal and serum samples. The R package caret (v6.0‐93) was used to perform partial least squares discriminant analysis and fit standard PLS models for classification. The variable of importance in projection (VIP) scores was generated to measure metabolites' importance in the PLS‐DA models, and dot plots were created to show the structural differences between groups. Statistical analysis was performed using Welch's *t*‐test. Spearman correlation analysis between gut microbiota and host metabolites was done by the R package psych (v2.2.9). Heatmaps, volcano plots, and dot plots were created with ggplot2 (v3.4.1).

### 6.8 Glucose tolerance test (GTT) and insulin tolerance test (ITT)

GTT or ITT tests were performed by measuring blood glucose levels with a Yuyue 306 using blood collected from the tail vein as described before [49, 51, 56, 59, 60].

### 6.9 H&E staining

Liver and adipose tissue were embedded in paraffin and sectioned. H&E staining was performed according to the standard protocol as described before [50, 51].

### 6.10 Probiotics culture and postbiotics preparation

Probiotics culture and postbiotics preparation were performed by following the protocol as described in a previous publication by following the standard protocol established in the lab [13].

### 6.11 Immunohistochemistry (IHC)

Paraffin‐embedded adipose tissues were sectioned, and the immunohistochemistry was performed according to the standard protocol established and reported in a previous study [51].

### 6.12 Statistical analysis

Data were presented as mean ± SEM. Statistical analyses were performed using GraphPad Prism 10. For comparisons between two groups, an unpaired Student's *t*‐test was used. For comparisons among groups involving two independent variables (Diet and Treatment), a two‐way ANOVA was performed, followed by Tukey's multiple comparisons test. For longitudinal data involving repeated measurements on the same animals over time (e.g., body weight changes, GTT, and ITT), a two‐way repeated measures ANOVA (or mixed‐effects model) was utilized to evaluate the main effects and interaction between time and treatment, followed by Holm–Šídák's or Tukey's multiple comparisons test. Statistical difference was presented as *p < 0.05, **p < 0.01, ***p < 0.001 or N.S. not significant.

## AUTHOR CONTRIBUTIONS

Ye Tian, Yifan Liu, and Yanbo Cheng performed the experiments and drafted the manuscript. Ye Tian, Liujing Huang, and Bingdong Liu analyzed the data and generated the figures. Xudong Mai and Yixin Chen developed and prepared the animal model for this study. Liwei Xie edited, proofed, and revised manuscript. All authors have read the final manuscript and approved it for publication.

## CONFLICT OF INTEREST STATEMENT

The authors declare no conflicts of interest.

## AI STATEMENT

Artificial intelligence‐assisted technologies (OpenAI‐GPT5.2 and Gemini 3.0) were used to polish the English writing in this manuscript and design the graphical abstract. We confirm that no AI tools were used in the processes of data generation, analyses, or visual content creation.

## ETHICS STATEMENT

All animal studies were approved by the Institute of Animal Care and Use Committee (IACUC) of the Institute of Microbiology, Guangdong Academy of Sciences (GDAS) (Permission #: GT‐IACUC202207011).

## Supporting information


**Figure S1.** Phylogenetic trees of key probiotic bacterial strains used for postbiotics preparation.
**Figure S2.** Postbiotics supplementation alters tissue distribution and metabolic parameters in diet‐induced obesity.
**Figure S3.** Metabolic characterization of HFD‐fed mice with or without postbiotics supplementation.
**Figure S4.** Postbiotics modify the structure of gastrointestinal flora and host metabolism under different dietary patterns.

**Table S1.** qPCR Primer list.

## Data Availability

All 16S rRNA gene amplicon sequencing data were deposited in the National Microbiology Data Center under the BioProjects NMDC10018300 (https://nmdc.cn/resource/genomics/project/detail/NMDC10018300). The raw data for 16S rRNA sequencing, cell‐free supernatant, serum, and fecal metabolomics analysis were deposited at GitHub at https://github.com/xielab2017/Postbiotics-improve-host-metabolic-syndrome-in-the-HFD-fed-mice. The code scripts used for data processing, analysis, and visualization have been deposited at GitHub for EasyMicroPlot (https://github.com/xielab2017/EasyMicroPlot) [54] and EasyMultiProfiler (https://github.com/liubingdong/EasyMultiProfiler) [55]. Supplementary materials (figures, tables, graphical abstract, slides, videos, Chinese translated version, and update materials) may be found in the online DOI or iMetaOmics http://www.imeta.science/imetaomics/.
