# Supplementary material for: Probiotics‐derived postbiotics improve host metabolic syndrome by activating the adipocyte thermogenic signaling pathway in high‐fat diet‐fed mice
Source: IMetaOmics. 2026 Mar 5;3(2):e70089. doi: 10.1002/imo2.70089 (PMC13674458; doi:10.1002/imo2.70089)
Supplement: Supplementary file 1 [file IMO2-3-e70089-s001.docx]

**SUPPORTING INFORMATION to**

**Probiotics-derived postbiotics improve host metabolic syndrome by activating the adipocyte thermogenic signaling pathway in high-fat diet-fed mice**

**Running Title**: Postbiotics improve host metabolic syndrome in high-fat diet-fed mice

Ye Tian^1,2,3,4#^, Liujing Huang^2#^, Yifan Liu^2^, Xudong Mai^2,3^, Yanbo Cheng^2^, Bingdong Liu^2^, Yixin Chen^2^, Liwei Xie^1,2,3,5*^

^1^Faculty of Synthetic Biology, Shenzhen University of Advanced Technology, Shenzhen, 518055, China

^2^State Key Laboratory of Applied Microbiology Southern China, Guangdong Provincial Key Laboratory of Microbial Culture Collection and Application, Guangdong Open Laboratory of Applied Microbiology, Institute of Microbiology, Guangdong Academy of Sciences, Guangzhou, 510070, China

^3^Department of Endocrinology and Metabolism, Zhujiang Hospital, Southern Medical University, Guangzhou, 510280, China

^4^Department of Rehabilitation, Shenzhen Children's Hospital, Shenzhen, Guangdong, 518000, China

^5^Fuyang Institute of Zhejiang Chinese Medical University, Hangzhou,311499, China

^#^These authors contributed equally: Ye Tian, and Liujing Huang

^*^Corresponding author: [xielw@gdim.cn](mailto:xielw@gdim.cn) (Liwei Xie)

**SUPPLEMENTARY FIGURES**

**Figure S1** Phylogenetic trees of key probiotic bacterial strains used for postbiotic preparation. (A) Phylogenetic tree of *Lactobacillus reuteri* strains, including the PLBK^®^1 and PLBK^®^2 strains used in this study, showing evolutionary relationships based on whole-genome sequence analysis. (B) Phylogenetic tree of *Lactobacillus gasseri* strains, including the PLBK^®^3 strain utilized for postbiotic preparation. (C) Phylogenetic tree of *Lactobacillus acidophilus* strains, highlighting the position of the PLBK^®^4 strain within the evolutionary context of this species. (D) Phylogenetic tree of *Bifidobacterium lactis* strains, including the PLBK^®^5 strain employed in this study, demonstrating its genetic relationship to other characterized strains.

**Figure S2** Postbiotic supplementation alters tissue distribution and metabolic parameters in diet-induced obesity. (A) Tissue-to-body weight ratios of interscapular brown adipose tissue (iBAT), inguinal white adipose tissue (iWAT), epididymal white adipose tissue (eWAT), and liver across experimental groups. Postbiotic treatment significantly reduced iWAT and eWAT mass in HFD-fed mice. (B) Area under the curve (AUC) analysis of glucose tolerance test (GTT) results, demonstrating significant improvement in glucose handling with postbiotic supplementation in both NCD and HFD-fed groups. (C) Area under the curve (AUC) analysis of insulin tolerance test (ITT) results, showing enhanced insulin sensitivity with postbiotic treatment in both dietary conditions. (D) Hepatic triglyceride (TG) content quantification revealing significant reduction in liver lipid accumulation with postbiotic supplementation in HFD-fed mice, but no effect in NCD-fed controls. (E) Gene ontology (GO) molecular function enrichment analysis of differentially expressed genes in Cluster I. Bubble plot shows significantly enriched terms related to lipid metabolism and extracellular matrix organization. Color intensity represents -log_2_(*p*-value), and bubble size corresponds to gene number. (F) KEGG pathway enrichment analysis of differentially expressed genes in Cluster I. Bubble plot highlights significant enrichment of TGFβ signaling, steroid biosynthesis, PPAR signaling, and ECM-receptor interaction pathways. Color intensity represents -log_2_(*p*-value), and bubble size corresponds to gene number. Data were presented as mean ± SEM. **p* < 0.05, ***p* < 0.01, ****p* < 0.001, N.S. indicates no significant difference.

**Figure S3** Metabolic characterization of HFD-fed mice with or without postbiotic supplementation. (A) Tissue-to-body weight ratios showing significant reduction in inguinal white adipose tissue (iWAT) and epididymal white adipose tissue (eWAT) mass with postbiotic treatment. (B) Body composition analysis showing lean and fat mass percentages. Postbiotic treatment showed a trend toward reduced fat mass (p = 0.0734). N.S. indicates no significant difference. (C-D) Area under the curve (AUC) analysis of glucose tolerance test (GTT) and insulin tolerance test (ITT) results, demonstrating significantly improved glucose homeostasis and insulin sensitivity with postbiotic supplementation. (E-K) Serum biochemical parameters: (E) Triglycerides (TG), (F) Total cholesterol (TCHO), (G) HDL-cholesterol (HDL-c), (H) LDL-cholesterol (LDL-c), (I) Non-esterified fatty acids (NEFA), (J) Aspartate aminotransferase (AST), (K) Alanine aminotransferase (ALT). Data were presented as mean ± SEM. ***p* < 0.01, ****p* < 0.001, N.S. indicates no significant difference.

**Figure S4** Postbiotics modify the structure of gastrointestinal flora and host metabolism under different dietary patterns. (A) PCA of NCD groups and HFD groups on the first two principal components was performed based on fecal microbiota at species level. Ellipses represent 50% and 95% CI. (B) PCA of PHFD groups on the first two principal components was performed based on small intestinal and fecal microbiota at species level. Ellipses represent 50% and 95% CI. (C and D) Partial Least Squares Discriminant Analysis (PLS-DA) of fecal metabolites and serum metabolites in the NCD groups (C) and HFD groups (D). Ellipses in PLS-DA represent a 95% CI. Statistical analysis was performed using Welch's t-test; n = 6 per group. (E and F) Relative abundances of phyla across the NCD groups and HFD groups (E); and PHFD groups (F). (G and H) The Variable of importance in projection (VIP) scores are generated to measure metabolites’ importance in the PLS-DA models. The postbiotics-related metabolites in fecal samples were identified according to VIP scores by comparing NCD_HF group versus NCD_PF group, and HFD_HF group versus HFD_PF group (G) in PLS-DA models. The postbiotics-related metabolites in serum samples were identified according to VIP scores by comparing NCD_PS group versus NCD_HS group, and HFD_PS group versus HFD_HS group (H) Triangles denote postbiotics-related metabolites with VIP scores over 1 in fecal samples (G) or serum samples (H) of both NCD and HFD PLS-DA models.
